# Hematopoietic Stem Cell Transplantation for Severe Combined Immunodeficiency (SCID)

**DOI:** 10.3389/fped.2019.00481

**Published:** 2019-11-19

**Authors:** Elie Haddad, Manfred Hoenig

**Affiliations:** ^1^CHU Sainte-Justine, Department of Pediatrics, Microbiology, Immunology and Infectious Diseases, University of Montreal, Montreal, QC, Canada; ^2^Department of Pediatrics, University Medical Center Ulm, Ulm, Germany

**Keywords:** severe combined immunodeficiency, SCID, clinical presentation, infections, hematopoietic stem cell transplantation, HSCT

## Abstract

Severe Combined Immunodeficiencies (SCID) are a heterogeneous group of monogenetic diseases. We describe the typical clinical presentation of patients with SCID as well as basic principles in diagnosis and therapy by hematopoietic stem cell transplantation. Therapeutic strategies may differ between subtypes and the inherent reduced capacity or inablility to reject a graft have to be considered.

## Introduction

SCID is defined by a severely impaired T-lymphocyte development responsible for the absence of adaptive immune response and profound T-lymphocytopenia. SCID diagnostic criteria proposed by the Primary immune Deficiency Treatment Consortium (PIDTC) help to distinguish typical SCID from leaky SCID and Omenn syndrome (Table 1) (1). There is a cohort of patients who do not fulfill the criteria given in Table 1 but can present with identical clinical features and have the same need for a rapid diagnosis and therapy. It is a matter of ongoing debate but not subject of this review whether these entities can be called “SCID.” As there is no other published definition for SCID this review will keep to the definition proposed by the PIDTC.

**Table 1:** SCID definition according to the primary immune deficiency treatment consortium published by Shearer et al. (1).

**Typical SCID**	**Leaky SCID or Atypical SCID**	**Omenn Syndrome**
Absence or very low number of T cells (CD3 T cells <300/μL) **AND** No or very low T-cell function (<10% of lower limit of normal) as measured by response to PHA **OR** T cells of maternal origin present	Reduced number of CD3 T cells for age up to 2 y, <1,000/μL for >2 up to 4 y, <800/μL for >4 y, <600/μL **AND** Absence of maternal engraftment **AND** <30% of lower limit of normal T-cell function (as measured by response to PHA)	Generalized skin rash **AND** Absence of maternal engraftment **AND** Detectable CD3 T cells, >300/μL **AND** Absent or low (<30% of normal) T-cell proliferation to antigens to which the patient had been exposed **OR** If the proliferation to antigen was not performed but at least 4 of the following 10 supportive criteria, at least 1 of which must be among those marked with an asterisk (*): Hepatomegaly Splenomegaly Lymphadenopathy Increased IgE level Increased absolute eosinophil count *Oligoclonal T cells measured by CDR3 length or flow cytometry * >80% of CD3^+^ or CD4^+^ T cells are CD45RO^+^ *Proliferation to PHA is reduced <30% of lower limit of normal *Proliferative response in mixed leukocyte reaction is reduced <30% of lower limit of normal *Mutation in SCID-causing gene

## Genetic Heterogeneity

SCID is a heterogeneous genetic disorder (see Table 2 for genes list). In the most recent prospective cohort of SCID in North America, only 7% of SCID patients don't show any mutation of known SCID-causing genes (3).

**Table 2:** Genetic SCID entities according to the IUIS classification (2).

**Immunophenotype**	**Gene affected**	**Phenotype MIM number**
B+NK–	*IL2RG*	300400
	*JAK3*	600802
B+NK+	*IL7R*	608971
	*PTPRC*	608971
	*CD3D*	615617
	*CD3E*	615615
	*CD3Z*	610163
	*CORO1A*	605000
	*LAT*	617514
B–NK+	*RAG1*	601457
	*RAG2*	601457
	*DCLRE1C*	602450
	*PRKDC*	615966
	*NHEJ1*	611290
	*LIG4*	608037
B–NK–	*AK2*	267500
	*ADA*	102700

### Immunophenotype

SCID entities are also classified by immunophenotype, depending on the presence of B-lymphocytes and NK cells, which are usually associated with specific gene defects (genotypes) (Table 2). This immunophenotypic categorization does not replace molecular identification, which is key for prognosis (4).

## Clinical Presentation

The clinical presentation of patients with SCID is variable. Usually, patients are asymptomatic at birth and present within their first weeks or months of life with infections and/or failure to thrive (FTT) (5). Some patients may also present with signs of immune dysregulation caused by autologous (Omenn syndrome) or allogeneic (maternal GvHD) T cells. Some genetic defects cause non-immunological and disease specific features. In the absence of allogenic hematopoietic stem cell transplantation (HSCT) or gene therapy (GT), patients ultimately die generally within the first year of life. Some patients may also present with leaky-SCID, usually caused by hypomorphic mutations in classical SCID causing-genes, responsible for a less severe phenotype with infections and autoimmunity.

## Recurrent and severe infections

Patients are classically not infected at birth, but in the absence of diagnosis, they will eventually present with severe, atypical and/or recurrent life threatening infections. Opportunistic infections can suggest a defect of specific cellular immunity but non-specific bacterial infections are also frequently observed in SCID patients (5). Airway infections due to viral (*Respiratory Syncytial Virus, Myxovirus, Adenovirus*) and opportunistic microorganisms such as *Pneumocystis jirovecii* (PJP) can cause severe pneumonias requiring mechanical ventilation. Intestinal infections with Rota-, Noro or Adenovirus can lead to severe chronic diarrhea. *Cytomegalovirus* (CMV) infection can be particularly severe and diffuse with pulmonary, liver, cardiac, intestinal, retinal, and/or central nervous system involvement.

Live vaccines with attenuated microorganisms, such as *Bacille Calmette-Guérin* (BCG) or *Rotavirus*, may lead to atypical infections and can have an unwanted diagnostic value. A chronic swelling or ulceration at the BCG injection site or a chronic diarrhea after oral Rotavirus vaccination should lead to further diagnostic steps and the formal exclusion of a T-lymphocyte deficiency.

## Specific Features Due to the Affected Gene

Patients with Adenosine-Deaminase (ADA)-deficiency may present with alveolar proteinosis (6) and/or bone abnormalities (cupping of osteochondral junctions) detectable on the chest radiograph. Some patients with DNA repair defects associated with SCID, such as Ligase-4 deficiency or Cernunnos/XLF may present with microcephalia and delayed neurological development.

## Signs of Immunodysregulation: Maternal Graft vs. Host Disease (GvHD) and Omenn Syndrome

T-lymphocytes transferred from the mother during pregnancy can proliferate, expand, and cause skin lesions and more rarely liver manifestations such as in GvHD following allogeneic HSCT (7). In most cases however, maternal T-lymphocytes are detected in patient's peripheral blood without any clinical signs. An increased risk for post-transplant acute GvHD has been demonstrated for patients with maternal T cells detectable at diagnosis (8).

In some cases, “hypomorphic mutations” of SCID-causing genes encode for proteins with some residual function, allowing for the development of few oligoclonal autoreactive T-lymphocytes, which proliferate and invade peripheral tissues and may be responsible for a so-called Omenn syndrome (definition see Table 1) with a generalized exanthema, lymphadenopathy, alopecia, hepato-/ splenomegaly, intractable diarrhea, and eosinophilia (9). Omenn syndrome and rarely maternal GvHD require immunosuppressive drugs.

## New-Born Screening by Quantification of T-cell Receptor Excision Circles (TREC)

Active infection and age > 3.5 months at HSCT are associated with poor survival after HSCT (4, 10, 11), underlining the importance of new-born screening (NBS) to diagnose patients as early as possible and to prevent infections (12, 13) (see review on Universal newborn screening for SCID). Screening programs have been currently established in all 50 US-states, 4 Canadian Provinces, Israel, Switzerland and Germany, and will be introduced in various European countries within next years.

## Basal and Specific Diagnostic Measures

In the absence of NBS, SCID should be suspected in presence of profound lymphopenia (14). However, blood cell count may have not been performed or the lymphopenia may be missed or absent (see below) and unfortunately, SCID diagnosis is then classically suspected on the clinical history of recurrent infections and/or failure to thrive (FTT). Present maternal T-lymphocytes or expansion of autoreactive autologous T-lymphocytes may lead to normal (or rarely increased) lymphocyte counts. Therefore, a normal lymphocyte count should not delay the analysis of lymphocyte subpopulations that is usually key for the diagnosis (1) and for the SCID categorization (see above). A lack of naïve T-lymphocytes (e.g., CD3+CD4+CD45RA+CCR7+) or more specifically of recent thymic emigrant cells (CD4+CD45RA+CD31+) is an important laboratory finding suggestive of thymic dysfunction. Lack of or diminished proliferation to PHA is considered an important diagnostic criterion (1), although it is sometimes difficult to perform when T-lymphocyte counts are very low. A skewed TCR-repertoire is a strong hint toward peripheral oligoclonal expansion of either maternal or autologous autoreactive T-lymphocytes.

Immunoglobulin levels should be interpreted with caution since IgG are of maternal origin during the first months of life, IgA can be physiologically absent, IgM can sometimes be normal, and increased IgE can be observed despite a complete absence of circulating B-lymphocytes and is a typical component of Omenn's syndrome.

Thoracic radiography can also be helpful to the diagnosis by showing, in addition to the absence of thymic shadow, characteristic features of ADA deficiency (15).

It is very important to look for and rule out a DiGeorge syndrome or any other thymic disorder, which can be responsible for a SICD phenotype, because the therapeutic strategy may involve thymic transplantation. The observation of the classical features associated with Del22q11 or CHARGE syndrome (for coloboma, heart defects, choanal atresia, growth retardation, genital, and ear abnormalities) together with a T-B+NK+ phenotype are important diagnostic criteria although it may be sometimes very difficult to distinguish primary thymic disorder from “classical” SCID in the absence of SCID-causing gene identification.

## Therapeutic Principles and Options

When SCID is suspected, the patient should be referred to an experienced center to perform the necessary diagnostic procedures (see above) and implement the first therapeutic measures as soon as possible (16). Infections have to be carefully investigated (including invasive measures such as biopsies or bronchoalveolar lavage) to provide specific therapy. In parallel, supportive care with Trimethoprim/sulfamethoxazole as prophylaxis for PJP, Ig replacement therapy, and patient isolation to avoid additional infections should be instituted. Some centers use Fluconazole to prevent candidiasis. Screening for CMV infection should be systematically performed (e.g., by a PCR in blood). Since breastfeeding is the major source of CMV transmission in SCID patients, many centers recommend to stop breastfeeding as soon as SCID diagnosis is suspected and allow it only when the mother is shown negative for CMV serology. There are considerable differences in institutional protocols for supportive care after diagnosis until HSCT for patients with SCID (17) and there is an urgent need for evidence-based strategies to limit patient infection.

HSCT or gene therapy (GT) (when feasible) represent the definitive treatment of SCID. In the absence of such therapy, patients usually die, except ADA-deficient SCID for which enzyme replacement by PEG-ADA may provide sufficient T-lymphocyte function for prolonged survival. Nevertheless, PEG-ADA is best given as a short bridging therapy before definitive treatment by HSCT or GT (18).

## HSCT for SCID

Overall survival after HCT was between 65 and 70% in big multicenter retrospective cohorts (4, 11, 19, 20) and between 85 and 90% in more recent prospective cohorts (21, 22). The factor with most impact on survival was the donor category with matched sibling donor (MSD) being better than all other donor categories (4, 11, 19, 20, 23). In a multivariate analysis including 571 patients with non-MSD HSCT, the variables associated with survival were: age and infection at HSCT, genotype, FTT, and race/ethnicity (4). Interestingly, in this study that was the first to assess the impact of genotype, survival in patients with *RAG* mutations was significantly better than in patients with *DCLRE1C* mutations (both being T-B-NK+ phenotypes).

The unique aspect of HSCT for SCID is that it can be performed without any conditioning regimen (CR). Nevertheless, the role of CR in HSCT for SCID remains very controversial. The theoretical advantages of the absence of CR are the absence of chemotherapy-induced toxicity and the lower incidence of GvHD, likely due to the absence of stress of recipient epithelial cells. However, in the absence of CR there is little chance for donor myeloid engraftment and only donor T-lymphocyte progenitors will engraft (or mature donor T-lymphocytes from the graft depending on the intensity of T-lymphocyte depletion). On the other hand, CR that contain myeloablative agents usually allow for stem cell engraftment, which is associated with a better T- and B-lymphocyte reconstitution (4, 11, 23–26). No significant difference in survival was observed when no-CR or immunosuppression (IS) only (IS-only) was compared with busulfan-containing reduced intensity (RIC) or myeloablative (MAC) in retrospective cohorts (4, 11, 20). However, RIC/MAC were associated with a better T- and B-lymphocyte reconstitution (4, 11) while no-CR/IS-only was associated with less acute GvHD (4). Most importantly, T- and B-lymphocyte reconstitution have been shown to be associated with a better long term prognosis (4, 11, 25, 27). Patients with absent (*RAG1, RAG2, DCLRE1C*) or non-functional B-lymphocytes (*JAK3, IL2RG*) usually need CR in order to have a normal B-lymphocyte function post-HSCT (28–30). In the context of active infection at transplantation, it has been shown that CR was associated with a poor survival (11). In this situation, a 2-step procedure has been proposed: a first HCT without CR and then, once the infection is cleared, a second HCT with CR to allow for stem cell engraftment.

It is logical to propose a CR for SCID patients with RAG and DCLRE1C deficiency because of residual NK cell function and in order to empty thymic and marrow niches that inhibit donor progenitor engraftment (25). However, the context of DCLRE1C deficit and other DNA-repair defects represent a very difficult situation since CR has been associated with severe long-term toxicity (31). For all those patients, a chemotherapy-free myeloablative strategy, such as anti-stem cell monoclonal antibody, are urgently needed (see review on “Conditioning Perspectives for Primary Immunodeficiencies.”

Usually, HSCT with a MSD are performed without CR with a survival >90%. However, in about 25% of cases (29), patients require a long-term Ig replacement therapy because of the absence of donor B-lymphocyte engraftment. Interestingly, more and more centers are using a form of reduced toxicity CR in MSD in order to induce donor B-lymphocyte engraftment to avoid this need for long-term Ig replacement therapy.

## Conclusion, Challenges, New Hopes

The first challenge is the assessment of the best CR (product, dosing) for patients diagnosed by NBS and planned to be transplanted at a young age, in order to reduce the chemotherapy-induced toxicity. A prospective randomized study assessing the best dose of Busulfan is presently ongoing in North America (CSIDE study, NCT03619551). There is also a new hope with the ongoing study testing anti-CD117 antibody as a non-toxic myeloablative agent (NCT02963064). Another challenge is the reduction of the rate of infection in patients diagnosed by NBS, which is still too high at 42% (22). There is still no consensus regarding the methods of isolation and infection prophylaxis for these patients, and studies assessing how to best prevent infections prior to HSCT are urgently needed. A further challenge is the lack of prospective and retrospective studies of HSCT for each given SCID genotype, in order to define the best strategy for each genotype. Clear-cut biomarkers to clearly define which patients need a re-transplantation in situation of poor T-lymphocyte reconstitution post-HSCT are also missing. Indeed, although CD4^+^ and CD4^+^CD45RA^+^ cell counts as soon as 6 months post-HSCT were shown to be statistically associated with long-term immune reconstitution and long-term survival (4), the majority of patients with low CD4^+^ and CD4^+^CD45RA^+^ cell counts still survived and displayed a good long-term immune reconstitution, making those criteria insufficient to decide to re-transplant or not for a given patient (26). Furthermore, clinical studies assessing the quality of life and the sequelae of very long-term (>20 years) survivors of HSCT for SCID are missing. The collaboration of North-American and European Consortium should allow to perform the majority of the above mentioned studies which require a sufficient number of patients.

The emergence of techniques for the genetic manipulation of autologous hematopoietic stem cells in the 1990s opened a novel therapeutic window for permanent cure of SCID patients. The major advantage of this strategy is the avoidance of side effects caused by allogeneic immunological reactions such as rejection or GvHD. For more information on this subject see the review “Autologous stem cell-based gene therapy for inherited disorders: state-of-the-art and future prospects.”

## Author Contributions

EH and MH have discussed and written the review article and have approved the final version.

### Conflict of Interest

The authors declare that the research was conducted in the absence of any commercial or financial relationships that could be construed as a potential conflict of interest.
